# Is the number of siblings associated with dietary patterns in adolescents? The 1993 birth cohort of Pelotas (Brazil)

**DOI:** 10.1371/journal.pone.0174087

**Published:** 2017-03-23

**Authors:** F. O. Meller, M. C. F. Assunção, A. A. Schäfer, C. Loret de Mola, D. L. Dahly, J. S. Vaz, F. C. Barros

**Affiliations:** 1 Post-Graduate Program in Epidemiology, Federal University of Pelotas, Pelotas, Brazil; 2 Department of Epidemiology and Public Health, University College Cork, Cork, Ireland; 3 Post-Graduate Program in Health and Behavior, Catholic University of Pelotas, Pelotas, Brazil; University of Oslo, NORWAY

## Abstract

Our study aimed to estimate the association between number of siblings and dietary patterns in adolescents. Prospective longitudinal study was developed using data from the birth cohort of the city of Pelotas, Brazil, which included 5249 participants. At the 18-year-old follow-up, from 4563 individuals located, 4106 were interviewed (follow-up rate 81.3%). Of these, 3751 were included in our principal component analysis of dietary patterns. Regular dietary intake of 45 food groups over the previous year was measured with a food frequency questionnaire. We identified four patterns, which accounted for 40% of the total variance in food group consumption. These were labeled “Protein and fast food”, “Fruit and vegetables”, “Common Brazilian”, and “Sweets, soft drinks, and dairy products”. Crude and adjusted analyses of the association between number of siblings and dietary patterns were performed using linear regression. The number of siblings was positively associated with a higher adherence to each dietary pattern, with the exception of the “Common Brazilian” patterns, for which there was no apparent relationship with number of siblings. The findings showed that a greater number of siblings is related to a more diverse diet in the later adolescence, which may predict better nutrient adequacy and health outcomes.

## Introduction

Dietary intake during the adolescence has an important role in both the prevention and treatment of diseases. Also, dietary patterns and habits acquired during adolescence are also likely to persist throughout adulthood [1].

Although dietary intake assessment is challenging [2], and the majority of studies focus on the intake of individual nutrients, foods or food groups [3], dietary patterns have been widely used in nutritional epidemiology to examine the joint effects of multiple dietary components [4]. This approach identifies overall patterns of food intake and their apparent effects on disease risks, allowing us to examine potential synergistic or antagonistic effects between nutrients and/or foods [4].

While previous studies have looked into the role of family structure on dietary intake [5–7], few have evaluated the effect of number of siblings on adolescent dietary patterns [8–10]. One of these studies found that individuals with no siblings had a higher daily intakes of nutrients (except carbohydrate and added sugars) compared to individuals with siblings, suggesting that only-children may have a better diet quality [9]. Other studies found that having more than one sibling is associated with higher nutritional risk (according to nutrients intake based on Reference Nutrient Intake) [10], and that additional siblings may decrease the availability of food for each child [8].

Considering the scarce literature on this subject, the aim of the present study was to examine the association between number of siblings and dietary patterns in 18 year-old adolescents from a birth cohort in Southern Brazil.

## Materials and methods

### Study design and sample

Pelotas is a city in the extreme south of Brazil, near the border of Uruguay, and it currently has approximately 343,000 urban inhabitants. Its main economic activities are rice production, commerce and education. All 1993 hospital-born infants (N = 5249, accounting for over 99% of all city births) whose families lived in urban Pelotas, and agreed to participate, were recruited for a birth cohort study. Cohort members were followed-up at 11, 15 and 18 years of age (in 2004, 2008, and 2011, respectively). The methods of the 1993 Pelotas (Brazil) Birth Cohort Study are published elsewhere [11, 12]. For this study we included individuals with information on the exposure at age 15 years (number of siblings) and on the outcome at 18 years (dietary pattern).

### Dietary patterns assessment

A semi-quantitative self-reported food frequency questionnaire (FFQ) available in electronic format was administered to adolescents at 18 years of age [13]. This FFQ is an adapted version of the most commonly used FFQ in Brazil [13, 14].

This instrument measures the consumption of 88 food items in the previous 12 months. Response options for each food item were: never or less than once/month, 1 to 3 times/month, once/week, 2 to 4 times/week, 5 to 6 times/week, once/day, 2 to 4 times/day, 5 times/day or more.

Food items were collapsed into 45 foods groups according to their nutritional characteristics (S1 Fig), and a principal component analysis (PCA) was performed to identify sets of food groups with correlated intake levels [6, 15].

We considered representative of each component all food items with a factor loading greater than 0.25. The Kaiser-Meyer-Olkin test and Bartlett’s test of sphericity were applied to verify whether the PCA assumptions were all met. Varimax rotation was applied to obtain components with a near zero or maximum loading to improve component interpretability. The number of components was based on screeplot of components with eigenvalues greater than 1. Identified dietary patterns were standardised as z-scores and assessed as continuous variables.

### Number of siblings

Mothers were asked, “*How many times have you ever been pregnant including this pregnancy*?” at baseline, and “*Have you had any children after [the child enrolled in the birth cohort]*?” and “*If yes*, *how many*?” at the 15-year-old follow-up. Using these questions we calculated the total number of siblings. The questions included live births only. The main exposure used in the analyses was number of siblings, which had four categories (0; 1; 2; ≥3).

### Analytical methods

For descriptive analysis of the variables studied, we presented absolute and relative frequencies of categorical variables, and measures of central tendency and dispersion of continuous variables.

Crude and adjusted analyses of the association between the number of siblings and dietary pattern scores were performed using linear regression. Adjusted models included the following variables to account for potential confounding: family income (in real), maternal education (in completed years), presence of the father (yes/no), maternal skin colour (white/black/other), and maternal age (in years).

Additionally, linear regression analyses were performed to assess the association between number of siblings and energy intake (Kcal) using FFQ data. Adjusted analysis included the variables listed above.

Regression results are reported as point estimates and corresponding 95% confidence intervals.

All statistical analyses were conducted using STATA 12.1.

### Ethical considerations

The present study was approved by the Ethics Committee of the Medicine School of the Federal University of Pelotas in the official letter numbered 05/11. Adolescents signed the term of free and informed consent in both follow-ups.

## Results

### Sample characteristics

Of the 5249 infants enrolled into the study at birth, 4563 were located. Of those, 127 refused to participate in the study and 330 were considered losses, totalizing 4106 interviewed at 18 years of age. Added to those known to have died, this represents an 81.3% follow-up rate. Of these, 4052 adolescents had complete data on dietary patterns. After excluding those with inaccurate reports of energy intake (<-2 and >+2 SD), following standard procedures [16], our final sample consisted of 3751 individuals.

There was no difference between the participants included in our analyses and those excluded in relation to the variables studied (number of total siblings, family income, maternal education, presence of the father, maternal skin colour, and maternal age at birth) (data not shown in Table).

Table 1 describes the characteristics of our sample. Most adolescents had the father living in the house when they were born (88.4%) and about one-third of them at the age of 15 had one sibling (31.3%). Regarding the mothers, the average education at birth was near 7 years (SD 3.5) and age at birth was 26 years (SD 6.4). In addition, median family income was 830 reais (interquartile range 420–1500).

**Table 1:** Characteristics of sample according to the variables studied (n = 3 751). The 1993 Pelotas Birth Cohort, Brazil.

**Variables**	**n**	**%**	
**Number of siblings**			
0	437	12.2	
1	1 119	31.3	
2	882	24.7	
≥3	1 137	31.8	
**Maternal skin color**			
White	2 907	77.5	
Black	676	18.0	
Other	167	4.5	
**Presence of the father**			
No	436	11.6	
Yes	3 315	88.4	
	**n**	**Mean**	**SD**
**Maternal education (completed years)**	3 745	6.8	3.5
**Maternal age (years)**	3 750	26.2	6.4
	**n**	**Median**	**IQR**
**Family income (real)**	3 591	830	420–1500

Maximum percentage of unknown observations: (n = 176; 4.7%) for the number of siblings variable.

SD: Standard deviation

IQR: Interquartile range

### Dietary patterns

We identified four dietary patterns, which together explained 40% of the total variance in food group consumption. The first pattern, which we called *protein and fast food*, included fish, processed meat (canned tuna/sardine, salt-cured meat, bacon), hamburger, hot dogs, viscera (heart and liver), fried and roasted chicken, and pork meat. The second pattern, *fruits and vegetables*, included banana, orange and tangerine, tomato, other fruits, vegetables and legumes. The third one, *common Brazilian*, included coffee, black beans, white sugar, fat (butter and margarine), white rice, and white bread. Finally, the fourth pattern, called *sweets*, *soft drinks*, *and dairy products*, included chocolate powder, regular sodas, dairy products, sweets, candies and caramels, and ice cream. All food items showed positive loadings (Table 2).

**Table 2:** Four components of dietary pattern of adolescents at 18 years of age (The 1993 Pelotas Birth Cohort, Brazil).

Dietary patterns	Explained variance	Food group	Loading
Protein and fast food	14.6%	Fish and shrimp	0.3153
		Processed meet (canned tuna/sardine, salt-cured meat, bacon)	0.3135
		Pizza	0.2999
		Hot dog and hamburger	0.2898
		Viscera (heart and liver)	0.2853
		Fried and roasted chicken	0.2715
		Pork meat	0.2581
Fruits and vegetables	10.4%	Banana	0.3512
		Orange and tangerine	0.3481
		Other fruits	0.3591
		Tomato	0.3079
		Vegetables and legumes	0.3029
		Other vegetables	0.2704
Common Brazilian	7.7%	Coffee	0.3977
		Black beans	0.3568
		White sugar	0.3901
		Fat (butter and margarine)	0.3513
		White rice	0.2687
		White bread	0.2516
Sweets, soft drinks, and dairy products	7.5%	Chocolate powder	0.3786
		Regular sodas	0.3303
		Dairy products (milk, yogurt, cheese)	0.3174
		Sweets (pudding/desserts and chocolate bar)	0.2906
		Candies/caramels	0.2784
		Ice cream	0.2639

### Regression models

Crude and adjusted analyses evaluating the association between number of siblings and dietary pattern scores are presented in Table 3. S2 Fig also shows the crude analyses. Adjusted models showed that individuals with three or more siblings, compared to only-children, had higher adherence to the *fruits and vegetables* pattern (β = 0.21 z-scores, 95% CI 0.12; 0.29); the *sweets*, *soft drinks*, *and dairy products* pattern (β = 0.40 z-scores, 95% CI 0.30; 0.50); and the *protein and fast food* pattern (β = 0.10 z-scores, 95% CI 0.05; 0.15). There was no difference in consumption of the *common Brazilian* pattern according to number of siblings. There was some mild attenuation of effect sizes in the adjusted models, but the results were qualitatively similar to those from the unadjusted models.

**Table 3:** Crude and adjusted analyses of association between number of siblings and dietary patterns (in z-score) (The 1993 Pelotas Birth Cohort, Brazil).

	Protein and fast food		Fruits and vegetables		Common Brazilian		Sweets, soft drinks, and dairy products	
	β (95% CI)	p value	β (95% CI)	p value	β (95% CI)	p value	β (95% CI)	p value
***Crude analysis***								
Number of total siblings		<0.001		<0.001^†^		0.269		<0.001^†^
0	Reference		Reference		Reference		Reference	
1	-0.02 (-0.07;0.03)		0.04 (-0.05;0.12)		-0.04 (-0.12;0.05)		0.06 (-0.04;0.15)	
2	0.05 (0.001;0.10)		0.16 (0.07;0.24)		0.03 (-0.06;0.12)		0.32 (0.22;0.43)	
≥3	0.13 (0.09;0.18)		0.25 (0.17;0.33)		0.003 (-0.08;0.09)		0.56 (0.46;0.66)	
Adjusted analysis*								
Number of total siblings		<0.001^†^		<0.001^†^		0.101		<0.001^†^
0	Reference		Reference		Reference		Reference	
1	-0.03 (-0.07;0.02)		0.04 (-0.04;0.12)		-0.03 (-0.12;0.06)		0.06 (-0.04;0.15)	
2	0.04 (-0,01;0.09)		0.14 (0.05;0.23)		0.05 (-0.04;0.15)		0.25 (0.16;0.36)	
≥3	0.10 (0.05;0.15)		0.21 (0.12;0.29)		0.03 (-0.06;0.12)		0.40 (0.30;0.50)	

*Adjusted for confounding variables: family income, maternal education, presence of the father, maternal skin colour, and maternal age at birth.

^†^linear trend test

Additional analyses were performed to assess the association between number of siblings and energy intake. In the adjusted analysis, adolescents with three or more siblings showed an increase of 666.7 Kcal (95% CI 454.5; 878.9) in energy intake compared to those who had no siblings (data not shown).

## Discussion

Our findings showed the number of siblings was positively associated with higher adherence to each dietary pattern except the *common Brazilian* pattern. Corroborating the results of the present study, a British study reported no association between number of siblings (older or younger) and ‘traditional’ diet (based on meat, potatoes and vegetables) at age 7 years. In addition, the authors found that ‘health-conscious’ dietary component (vegetarian style foods, rice, pasta, salad and fruit) was positively associated with the number of older siblings the child had, whereas the ‘junk’ diet component (high-fat and sugar content, processed and convenience foods) was positively associated with the total number of siblings (older or younger) [17]. Similarly, a study carried out in Spain found that having more than one sibling was related to higher nutritional risk [10]. Such finding may be associated with less attention being given to food or individuals in families with a larger number of children [18].

Two previous studies have found that a higher number of siblings was associated with lower average daily intake of total protein, animal protein, calcium, vitamins, and fat; but a higher consumption of carbohydrate and added sugar [9, 19]. The finding that individuals with siblings had a lower intake of all nutrients except carbohydrate and added sugar indicates that they have diets that rely on cheaper nutrients as sources of energy rather than more expensive diets including proteins and fats [19]. One possible explanation is that the greater number of siblings means that the family has less income to spend per family member [20].

Our results showed that a greater number of siblings was associated with a more diverse diet. This finding may be reflecting the nutritional transition occurring in lower and middle income countries [21, 22]. Study including children from eight European countries reported that those from lower socioeconomic status (SES) have higher risk of unhealthy eating [23]. Also, recent review with studies from low and middle-income countries (the majority of them were conducted in Brazil) including adults showed that higher SES was associated with a healthier dietary pattern [24]. Since we observed adherence to varied patterns among individuals with siblings, it may suggest that these adolescents are in the middle of a nutritional transition (i.e. going from a dietary pattern typical from low-income countries, where higher SES is positively associated to unhealthier patterns, to a dietary pattern typical from high-income countries, where higher SES is positively associated to healthier patterns) [25].

A greater dietary variety may predict better nutrient adequacy and health outcomes [26, 27]. Also, it is important to note that dietary variety, food group intakes, and nutrient adequacy are all strongly correlated with energy intake [27].

Some methodological considerations should be highlighted. Due to loss of follow-up, potential selection biases might exist, however we compared the sample participants with the original participants assessed at the baseline and observed no difference in relation to the variables included in our analysis (data not shown). The 12-month recall period for the FFQ may be considered a limitation of our study, since it may generate recall bias. While we adjusted our regression models for a number of potential confounding factors, we cannot exclude the possibility that residual confounding explains the associations we have described. It is also important to emphasize that adherence to a dietary pattern does not, necessarily, exclude the adherence to other patterns, and the dietary patterns we identified may be not generalizable to other populations [28]. Finally, we used the information on the number of siblings at 15-year-old follow-up because we did not have this information at 18 year-old follow-up. Moreover, we do not have the information whether the siblings live together with the adolescent enrolled in the cohort.

Some strengths of this study are its high follow-up rate and its longitudinal design. Also, the four independent dietary components identified in our study explained 40% of the variation in adolescent food group consumption. In other studies which used PCA to analyze dietary patterns, the variance ranged from 15% to 31% [6, 15, 29].

In conclusion, our results showed that the number of siblings is associated with the dietary patterns in later adolescence, even after adjustment for potential confounders. Having siblings is associated to higher adherence to the *protein and fast food* pattern; the *fruits and vegetables* pattern; and the *sweets*, *soft drinks*, *and dairy products* pattern, which characterize a diversified diet.

## Supporting information

S1 Fig Forty-five food items of the food frequency questionnaire categorized according to nutritional characteristics (The 1993 Pelotas Birth Cohort, Brazil). (DOCX)

S2 Fig Crude analyses of association between number of siblings and dietary patterns (in z-score) (The 1993 Pelotas Birth Cohort, Brazil). (DOCX)
