# Supplementary material for: Is the number of siblings associated with dietary patterns in adolescents? The 1993 birth cohort of Pelotas (Brazil)
Source: PLoS One. 2017 Mar 23;12(3):e0174087. doi: 10.1371/journal.pone.0174087 (PMC5363840; doi:10.1371/journal.pone.0174087)
Supplement: S1 Fig — (DOCX) [file pone.0174087.s001.docx]

**S1 Fig. Forty-five food items of the food frequency questionnaire categorized according to nutritional characteristics (The 1993 Pelotas Birth Cohort, Brazil).**

| **Food items** | |  | |  |
| --- | --- | --- | --- | --- |
| 1 | Rice | 24 | Pork meat |  |
| 2 | Black beans | 25 | Pizza |  |
| 3 | Coffee | 26 | Fish and shrimp |  |
| 4 | Sugar | 27 | Processed meats (canned tuna/sardine, salt-cured meat, bacon) |  |
| 5 | Vegetables spices (garlic, onion, sweet pepper) | 28 | Ham, mortadella and sausage |  |
| 6 | Homemade bread | 29 | Roast and fried chicken |  |
| 7 | Artificial fruit juice | 30 | Viscera (heart, liver) |  |
| 8 | Vegetables and legumes (cabbage, lettuce, chayote, cucumber, green beans, beetroot, cauliflower, lentil, pea, chickpea) | 31 | Fruits (papaya, pineapple, avocado, mango, peach, guava, pear, apple, watermelon or melon, strawberry, grape) |  |
| 9 | Quibe | 32 | Red meat (steak and t-bone beef) |  |
| 10 | Orange vegetables (carrot, pumpkin) | 33 | Dairy products (milk, yogurt, cheese, soft cheese) |  |
| 11 | Orange or tangerine | 34 | Chocolate powder |  |
| 12 | Banana | 35 | White bread |  |
| 13 | Fresh juice | 36 | Mayonnaise |  |
| 14 | Tomato | 37 | Egg |  |
| 15 | Snacks (chips and salty crackers) | 38 | Pasta, fried polenta and corn |  |
| 16 | Sweet cookies | 39 | Fat (butter and margarine) |  |
| 17 | French fries | 40 | Regular sodas |  |
| 18 | Popcorn | 41 | Light sodas |  |
| 19 | Sweets (pudding/desserts, chocolate bar) | 42 | “Mate drink” | |
| 20 | Cakes | 43 | Potato and cassava | |
| 21 | Ice cream | 44 | Canned vegetables |  |
| 22 | Candies/caramels | 45 | Whole bread |  |
| 23 | Fast foods (hamburger, hot dog) |  |  |  |
