# Supplementary material for: Is the number of siblings associated with dietary patterns in adolescents? The 1993 birth cohort of Pelotas (Brazil)
Source: PLoS One. 2017 Mar 23;12(3):e0174087. doi: 10.1371/journal.pone.0174087 (PMC5363840; doi:10.1371/journal.pone.0174087)

**S2 Fig. Crude analyses of association between number of siblings and dietary patterns (in z-score) (The 1993 Pelotas Birth Cohort, Brazil).**


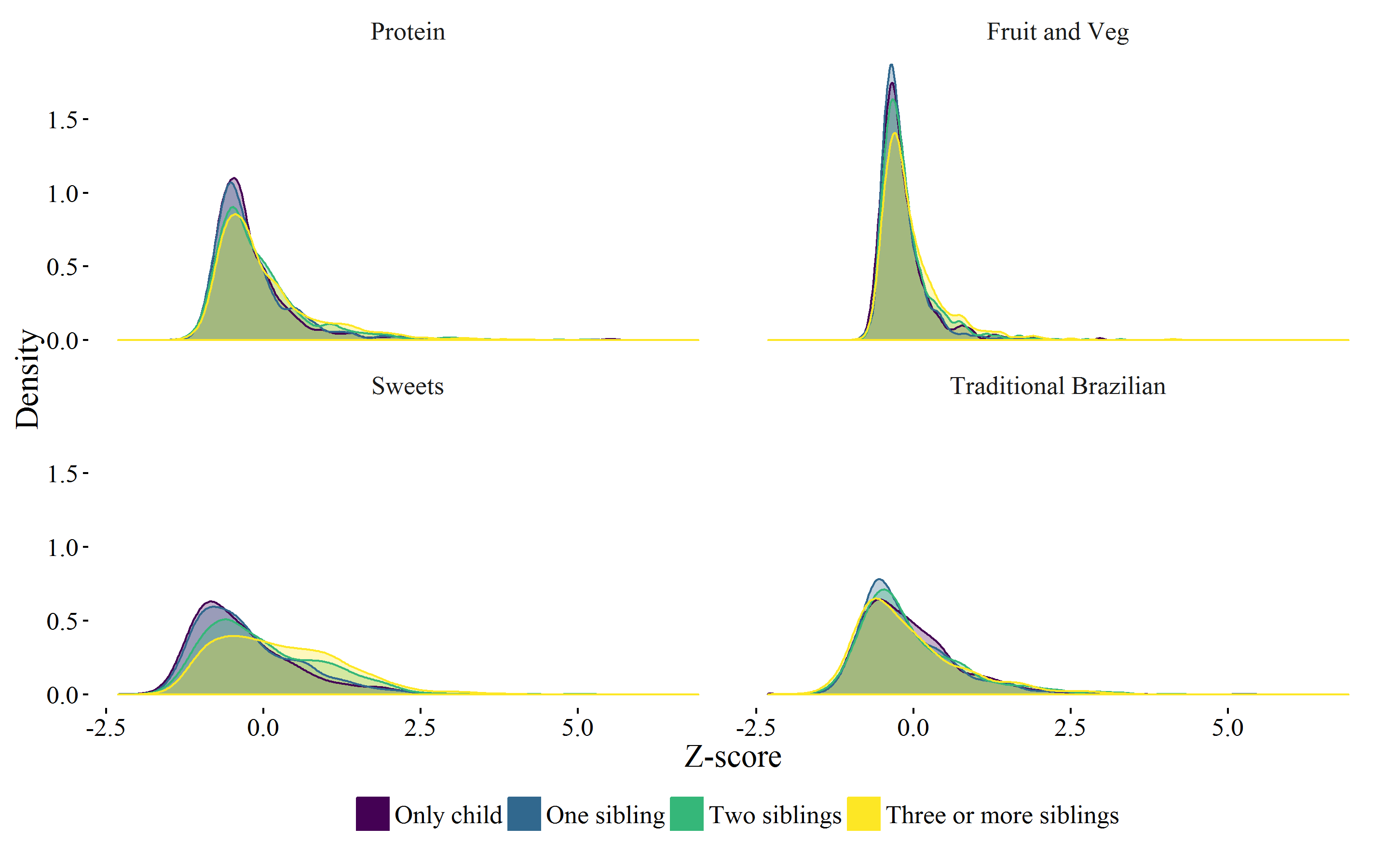

Supplement: S2 Fig — (DOCX) [file pone.0174087.s002.docx]
